# A novel *GWT1* A397T substitution combined with *TAC1B* F214I reduces manogepix susceptibility and fosmanogepix efficacy in *Candida auris*

**DOI:** 10.1128/spectrum.02078-26

**Published:** 2026-09-01

**Authors:** Tatsuro Hirayama, Natsume Kitahori, Mizuki Ishimine, NaNa Nakada, Yuya Ito, Naoki Iwanaga, Takahiro Takazono, Taiga Miyazaki, Koichi Izumikawa, Katsunori Yanagihara, Koichi Makimura, Kazuhiro Tsukamoto, Hiroshi Mukae

**Affiliations:** 1 Department of Pharmacotherapeutics, Nagasaki University Graduate School of Biomedical Sciences 200674, Nagasaki, Japan; 2 Department of Infectious Diseases, Nagasaki University Graduate School of Biomedical Sciences 200674, Nagasaki, Japan; 3 Department of Respiratory Medicine, Nagasaki University Hospital 88380, Nagasaki, Japan; 4 Division of Respirology, Rheumatology, Infectious Diseases, and Neurology, Department of Internal Medicine, Faculty of Medicine, University of Miyazaki 12951, Miyazaki, Japan; 5 Department of Laboratory Medicine, Nagasaki University Hospital 88380, Nagasaki, Japan; 6 Teikyo University Institute of Medical Mycology, Teikyo University 13094, Itabashi City, Tokyo, Japan; Debreceni Egyetem, Debrecen, Hungary

**Keywords:** TAC1B, GWT1, manogepix, fosmanogepix, *Candida auris*

## Abstract

Fosmanogepix (FMGX) is the prodrug of manogepix (MGX), which targets Gwt1, an inositol acyltransferase encoded by *GWT1* and required for glycosylphosphatidylinositol anchor biosynthesis. To identify genetic changes associated with reduced MGX susceptibility in *Candida auris*, we exposed strain NCPF8971 to MGX. A stable isolate with an MGX minimum inhibitory concentration (MIC) of 1.0 µg/mL compared with 0.03 µg/mL for the parental strain carried *GWT1* A397T and *TAC1B* F214I substitutions. Reciprocal CRISPR-Cas9 editing demonstrated that *GWT1* A397T, *TAC1B* F214I, and the combined substitutions increased the MIC to 0.06, 0.5, and 1.0 µg/mL, respectively; reversion restored the MIC stepwise to the parental level. In a *Galleria mellonella* infection model, FMGX improved survival and reduced fungal burden in larvae infected with the parental strain, whereas these effects were attenuated or absent in larvae infected with the engineered mutants. These findings identify *GWT1* A397T as a modest contributor to reduced MGX susceptibility and show that this target alteration further increases the MIC in a *TAC1B* F214I background. The combined genotype was associated with a diminished FMGX treatment response in this invertebrate model.

**IMPORTANCE:**

*Candida auris* is an emerging yeast that can resist several antifungal drugs and cause difficult-to-treat invasive infections. Fosmanogepix is an antifungal agent that acts on a target different from those of existing drugs. We investigated how *C. auris* could become less susceptible to fosmanogepix and whether the responsible genetic changes would affect treatment in an infection model. We found that a change in the protein targeted by the drug modestly reduced susceptibility, whereas a change in a regulatory protein previously linked to increased activity of a drug efflux pump caused a greater reduction; the combination of both changes further weakened the drug’s activity. The combined changes were also associated with a reduced response to fosmanogepix in an insect infection model. These findings show that changes affecting the drug target and drug efflux may act together to reduce antifungal activity and support monitoring for multiple pathways of reduced susceptibility.

## OBSERVATION

*Candida auris* represents an emerging multidrug-resistant yeast, necessitating the development of new antifungal agents (1). Fosmanogepix (FMGX) is a prodrug of manogepix (MGX), which inhibits the inositol acyltransferase Gwt1, an essential enzyme in glycosylphosphatidylinositol anchor biosynthesis (2, 3). MGX exhibits potent *in vitro* activity against multidrug-resistant *C. auris* (4), and FMGX has demonstrated clinical activity in *C. auris* candidemia (5). As MGX/FMGX resistance could emerge under drug exposure, defining the related genetic determinants and their *in vivo* impact is important.

To generate MGX-reduced-susceptibility derivatives, we plated *C. auris* NCPF8971 (clade I) (6) onto Sabouraud dextrose agar containing MGX at four times the parental MIC, incubated the culture at 30°C for 3 days, and determined the MICs using CLSI M27 broth microdilution (50% growth inhibition endpoint) (7). Of the 94 recovered colonies, 13 initially exhibited an MGX MIC of 0.5 µg/mL. After serial passage on drug-free medium, one isolate (8971R) retained reduced susceptibility, with an MIC of 1.0 µg/mL upon repeat testing, compared with 0.03 µg/mL for the parental strain (Table 1; Fig. S1). Targeted *GWT1* and *TAC1B* sequencing in 8971R identified two nonsynonymous substitutions: *GWT1* A397T and *TAC1B* F214I, respectively.

**TABLE 1:** MGX MICs of the parental strain, MGX-selected isolate, and engineered mutants (CLSI M27; 50% growth inhibition endpoint)

Strain	MGX MIC (µg/mL)
NCPF8971 (parental)	0.03
NCPF8971 *gwt1*^A397T^	0.06
NCPF8971 *tac1b*^F214I^	0.5
NCPF8971 *gwt1*^A397T^/*tac1b*^F214I^	1.0
8971R (MGX-selected)	1.0
8971R *gwt1*^T397A^	0.5
8971R *tac1b*^I214F^	0.06
8971R *gwt1*^T397A^/*tac1b*^I214F^	0.03

**Fig 1:**
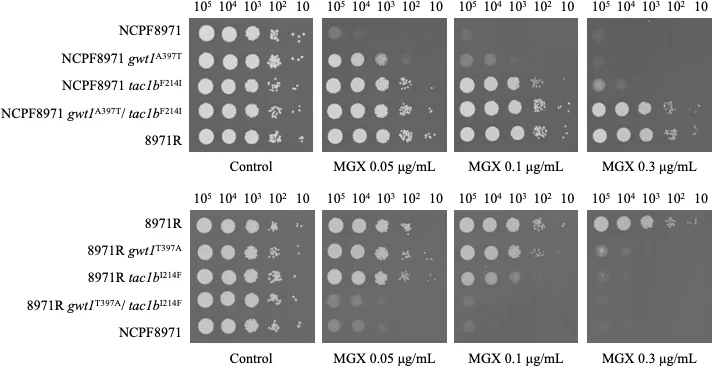
*GWT1* and *TAC1B* mutations reduced MGX susceptibility in *C. auris*. The effects of mutations in *GWT1* and *TAC1B* on MGX susceptibility were examined using a spot dilution assay. Log-phase cells were serially diluted and spotted onto SC agar plates containing the indicated MGX concentrations. The plates were incubated at 30°C for 48 h.

To test the contribution of each mutation, we used CRISPR-Cas9 ribonucleoprotein-mediated editing to introduce *GWT1* A397T and/or *TAC1B* F214I into the parental background and revert each mutation in 8971R (Fig. S2) (8, 9). *GWT1* A397T introduction increased the MGX MIC twofold (0.06 µg/mL), whereas *TAC1B* F214I increased the MIC to 0.5 µg/mL, and the double mutant reached 1.0 µg/mL (Table 1; Fig. S2). In contrast, reverting *GWT1* A397T and/or *TAC1B* F214I in 8971R reduced the MIC stepwise back to the parental level (Table 1; Fig. S1); spot dilution assays on MGX-containing agar were consistent with the MIC results (Fig. 1). These data indicate that the newly identified *GWT1* A397T substitution contributes to reduced MGX susceptibility and that concomitant *TAC1B* F214I further strengthens this phenotype.

*TAC1B* encodes a zinc-cluster transcription factor that regulates Cdr1, an ABC transporter, and a major azole resistance determinant in *C. auris* (10, 11). Notably, substitutions at Tac1b residue F214 (e.g., F214S and F214L) reportedly represent gain-of-function alterations associated with fluconazole resistance and increased *CDR1* expression (10, 11). We previously reported that the Tac1b–Cdr1 pathway contributed to reduced MGX susceptibility in *C. auris* (12). In the present study, introduction of *GWT1* A397T into the *TAC1B* F214I mutant background further increased the MGX MIC from 0.5 to 1.0 µg/mL. *CDR1* expression and efflux activity were not directly examined in the engineered strains. However, these findings suggest that a Gwt1 target alteration can further reduce MGX susceptibility in a genetic background carrying *TAC1B* F214I, which has previously been linked to Tac1b–Cdr1 pathway activation.

**Fig 2:**
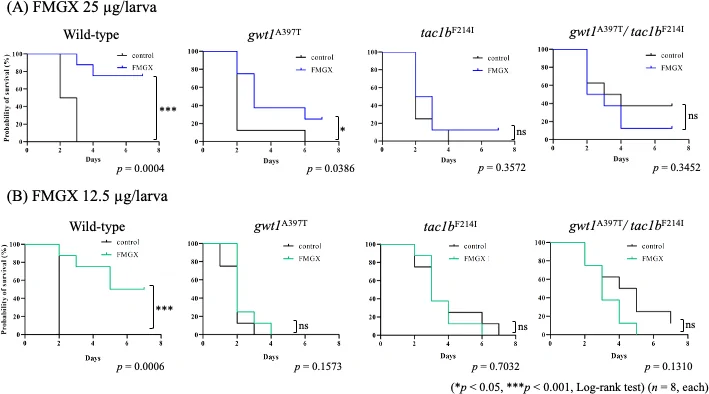
Fosmanogepix treatment effect in *Galleria mellonella* using survival curve analysis. (**A**) FMGX was administered at 25 µg per larva. (**B**) FMGX was administered at 12.5 µg per larva. Each experimental group consisted of eight larvae and was monitored for 7 days. Statistical significance was determined using the log-rank test (*P < 0.05; ***P < 0.001; ns, not significant).

**Fig 3:**
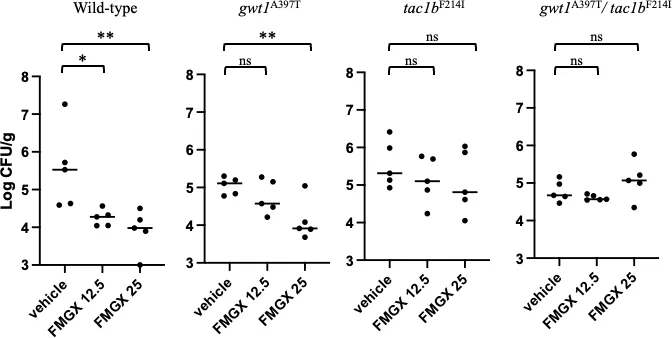
Fosmanogepix treatment effect in *Galleria mellonella* using fungal burden evaluation larvae received vehicle or FMGX at 12.5 or 25 µg per larva. Larvae were homogenized 2 days after infection and plated onto 0.2 G/L chloramphenicol-containing YPD agar; CFU per larva was enumerated after incubation at 30°C for 2 days. Each experimental group comprised five larvae. Statistical significance was determined using one-way ANOVA, followed by Dunnett’s multiple-comparison test (*P < 0.05; **P < 0.01; ns, not significant).

To evaluate whether these genotypes translated into altered FMGX efficacy *in vivo*, we used a *Galleria mellonella* infection model (13). We inoculated larvae with 10 µL of *C. auris* suspension (approximately 1 × 10^7^ to 5 × 10^7^ CFU/mL) into the left proleg. After incubation at 37°C for 2 h, each larva was injected into the right proleg with 10 µL of FMGX solution at 2.5 or 1.25 mg/mL, corresponding to 25 or 12.5 µg per larva, respectively. Based on a mean larval weight of approximately 0.30 g (approximate range, 0.25–0.35 g), these doses corresponded to approximately 83 mg/kg (range, 71–100 mg/kg) and 42 mg/kg (range, 36–50 mg/kg), respectively. The vehicle (10% DMSO; 40% PEG300; 5% Tween 80; 45% saline) was administered as the control treatment. No mortality was observed in uninfected larvae treated with FMGX at either 25 or 12.5 µg per larva during the 7-day observation period. Each survival group comprised eight larvae and was monitored for 7 days. Survival curves were compared using the log-rank test (Fig. 2). FMGX significantly improved the survival of larvae infected with the wild-type strain at both doses: 25 µg per larva (*P* = 0.0004) and 12.5 µg per larva (*P* = 0.0006). In contrast, the survival benefit was attenuated in the mutant groups: the *GWT1* A397T strain showed a significant benefit only at the relatively high dose of 25 µg per larva (*P* = 0.0386), whereas neither the *TAC1B* F214I strain nor the double mutant showed a significant survival benefit at either dose (Fig. 2). Consistent with the survival findings, FMGX significantly reduced the fungal burden on Day 2 post-infection in the wild-type strain at both doses, whereas the reductions remained limited or absent in the mutant strains (Fig. 3). To the best of our knowledge, this is the first study to evaluate FMGX treatment in a *Galleria* model of *C. auris* infection and link genotype-associated shifts in MGX MICs to a diminished FMGX treatment response in this model. Vehicle-treated larvae infected with the double mutant showed higher survival than those infected with the wild-type strain. This difference was statistically significant in the experiment evaluating the 12.5 µg per larva dose but not in the experiment evaluating the 25 µg per larva dose. Because Gwt1 is involved in glycosylphosphatidylinositol anchor biosynthesis and the localization of cell-surface proteins, alterations in Gwt1 function may potentially affect fungal fitness or virulence (2). Additionally, antifungal resistance-associated regulatory mutations may impose fitness costs (14). Thus, the relatively high survival of larvae infected with the double mutant may reflect an effect of *GWT1* A397T and/or *TAC1B* F214I on fungal fitness or virulence. However, this finding was not consistent between the two experiments, and the study was not designed specifically to compare virulence among strains. Therefore, dedicated experiments are required to confirm this possibility.

*GWT1* encodes an endoplasmic reticulum-localized inositol acyltransferase that catalyzes the inositol acylation of glucosamine-phosphatidylinositol (GlcN-PI), an early and essential step in glycosylphosphatidylinositol anchor biosynthesis (2). Consistent with this central role, Gwt1 substitutions have been associated with reduced susceptibility to MGX in other *Candida* species, including substitutions at a conserved valine residue that reproducibly shift MGX MICs (15). Recent cryo-electron microscopy structures have further revealed that manogepix occupies a hydrophobic cavity within the palmitoyl-CoA binding site of fungal Gwt1 and that resistance-associated substitutions cluster around the inhibitor-binding pocket (3). Although the structural impact of the *C. auris* Gwt1 A397T substitution remains to be defined, our genotype-phenotype-treatment linkage supports the hypothesis that Gwt1 variation could translate modest *in vitro* MIC shifts into a diminished FMGX treatment response in the *G. mellonella* infection model, thereby underscoring the need to monitor *GWT1* variants as FMGX use expands in the future. A limitation of this study was that whole-genome sequencing was not performed. Therefore, the presence of additional mutations in the MGX-selected isolate 8971R cannot be excluded.

In summary, we identified a previously unreported *GWT1* A397T substitution in *C. auris* selected under MGX pressure and demonstrated its contribution to reduced MGX susceptibility. Moreover, concomitant *TAC1B* F214I further increased the MGX MIC, and the combined genotype was associated with a diminished FMGX treatment response in the *G. mellonella* infection model. These findings support genomic surveillance for *GWT1* and *TAC1B* mutations during MGX/FMGX development and clinical use, emphasizing the value of *in vivo* models in assessing the therapeutic impact of emerging resistance determinants.

## Data Availability

All data generated or analyzed during this study are included in this published article and its supplemental materials.

## SUPPLEMENTAL MATERIAL

Tables S1 and S2 spectrum.02078-26-s0001.docx MICs of strains exposed to MGX and primer list.

Fig. S1 and S2 spectrum.02078-26-s0002.pdf Broth microdilution plates and schematic diagram of CRISPR-Cas9.

ASM does not own the copyrights to Supplemental Material that may be linked to, or accessed through, an article. The authors have granted ASM a non-exclusive, world-wide license to publish the Supplemental Material files. Please contact the corresponding author directly for reuse.
