# Supplementary material for: A novel GWT1 A397T substitution combined with TAC1B F214I reduces manogepix susceptibility and fosmanogepix efficacy in Candida auris
Source: Microbiol Spectr. 2026 Sep 1;14(10):e02078-26. doi: 10.1128/spectrum.02078-26 (PMC13688795; doi:10.1128/spectrum.02078-26)
Supplement: Supplementary file 1 [file spectrum.02078-26-s0002.pdf]

Figure S1

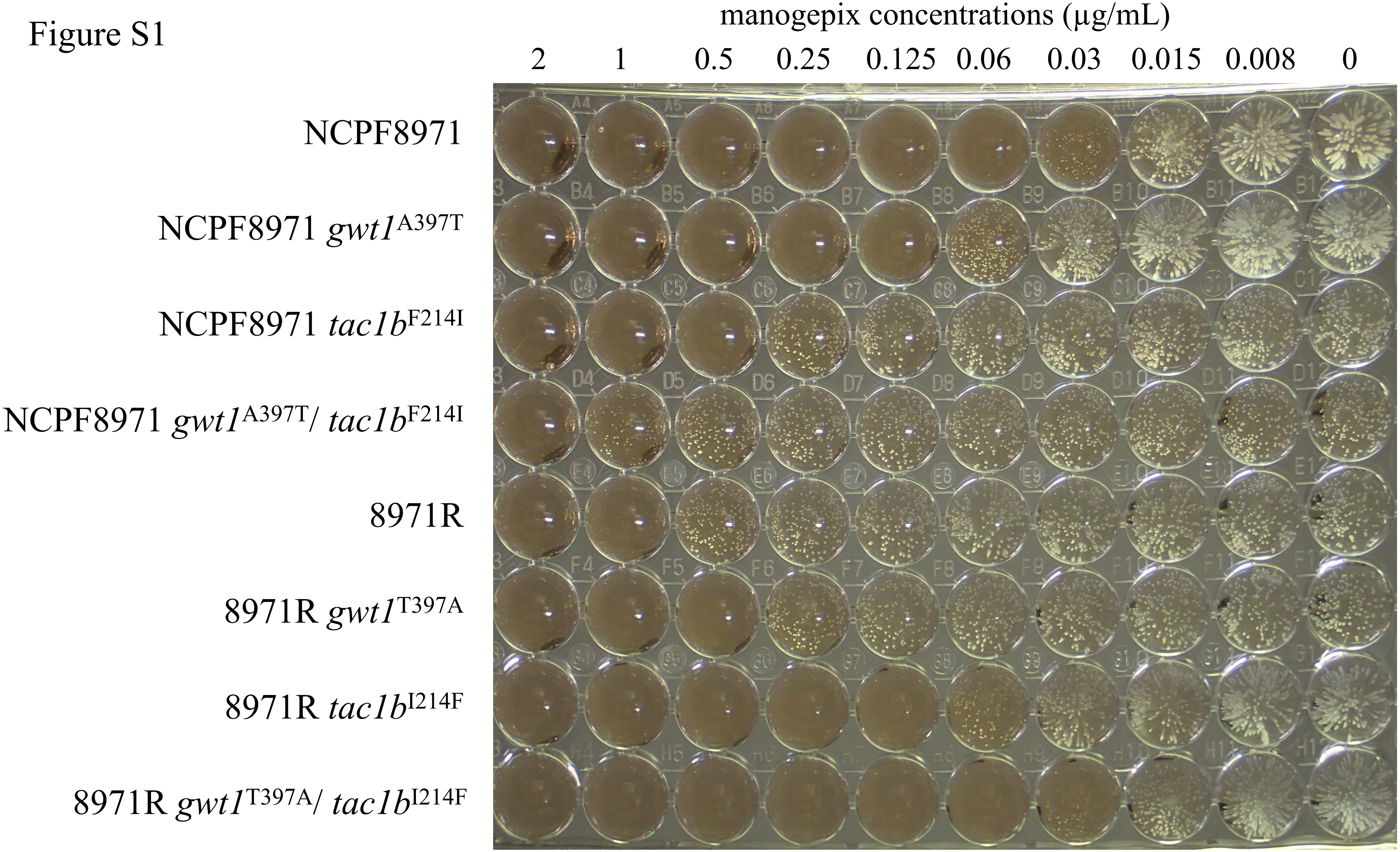

Supplementary Figure S1. Representative broth microdilution plates showing the manogepix MICs for the indicated *Candida auris* strains.

Representative broth microdilution plates showing the MGX MIC endpoints for the parental strain, the engineered *GWT1* A397T and *TAC1B* F214I single and double mutants, the MGX-selected isolate 8971R, and the corresponding revertant strains. MICs were determined according to the CLSI M27 broth microdilution method using a 50% growth inhibition endpoint. MGX concentrations are indicated above the wells.

Figure S2A

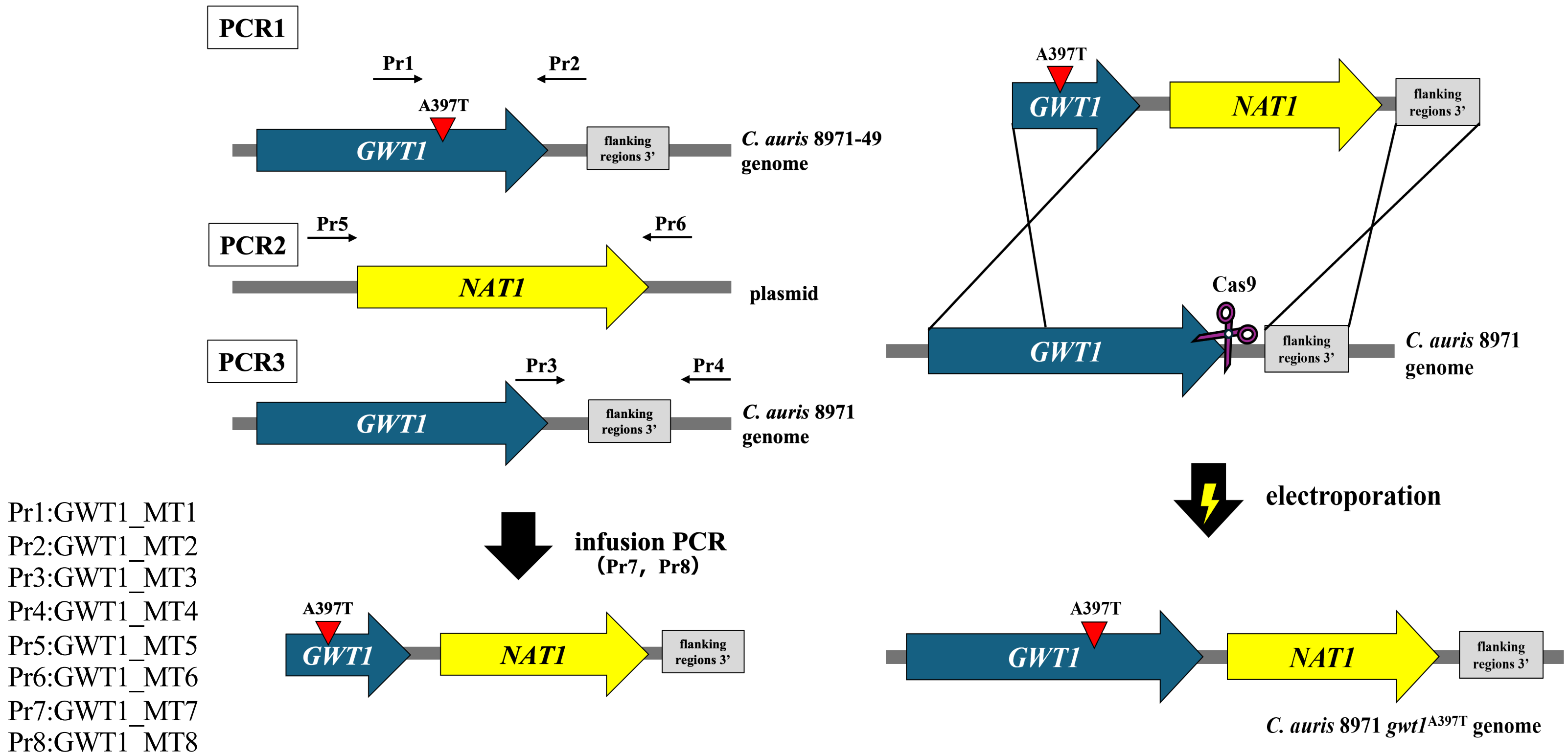

Supplementary Figure S2A. Introduction of the *GWT1* A397T substitution by CRISPR-Cas9. The left panel shows the infusion PCR strategy used to introduce the *GWT1* A397T substitution into the wild-type *GWT1* allele. The resulting repair construct contained the 3′ region of *GWT1* carrying the A397T substitution, the *NAT1* selection marker, and a downstream homologous region. The right panel shows CRISPR-Cas9-mediated introduction of the repair construct.

Figure S2B

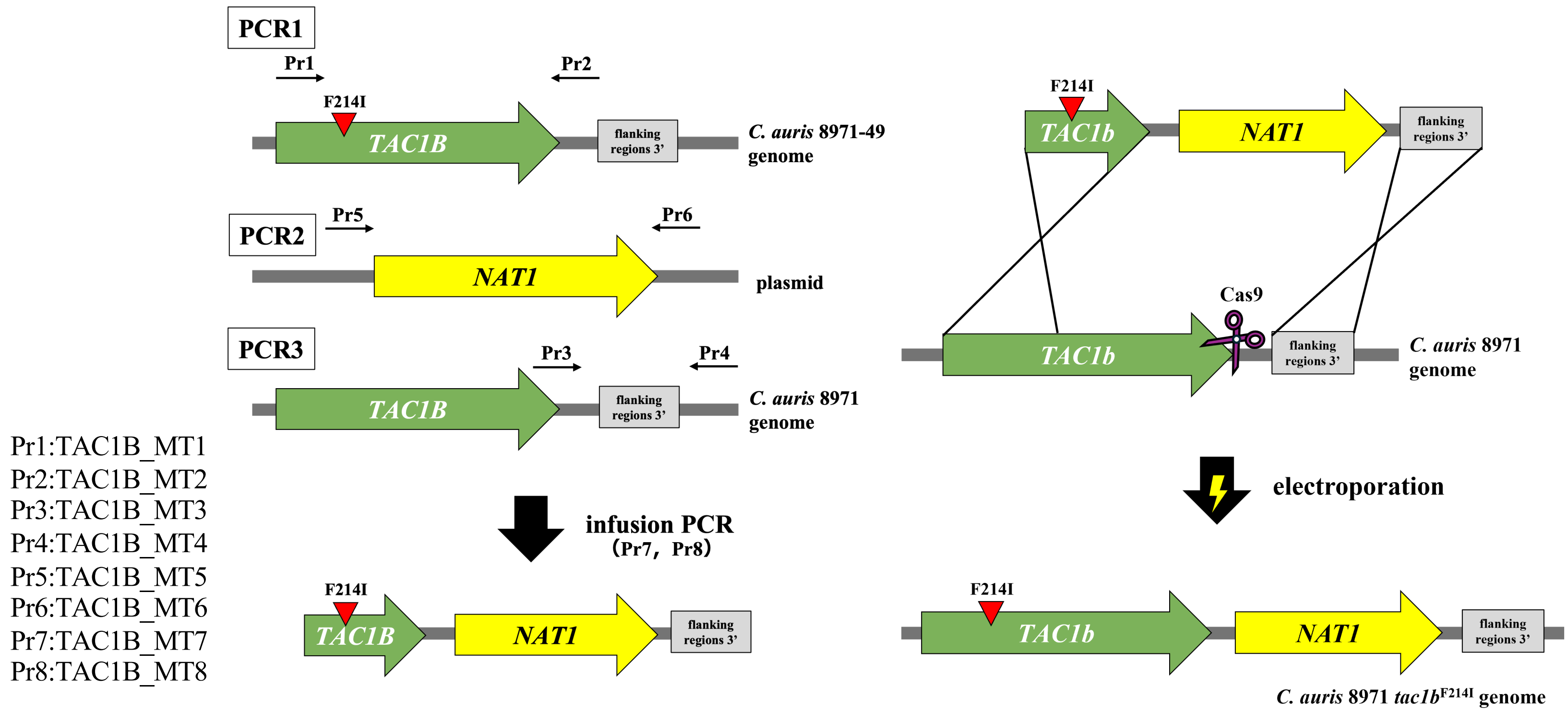

Supplementary Figure S2B. Introduction of the *TAC1B* F214I substitution by CRISPR-Cas9. The left panel shows the infusion PCR strategy used to introduce the *TAC1B* F214I substitution into the wild-type *TAC1B* allele. The resulting repair construct contained the 3′ region of *TAC1B* carrying the F214I substitution, the *NAT1* selection marker, and a downstream homologous region. The right panel shows CRISPR-Cas9-mediated introduction of the repair construct.

Figure S2C

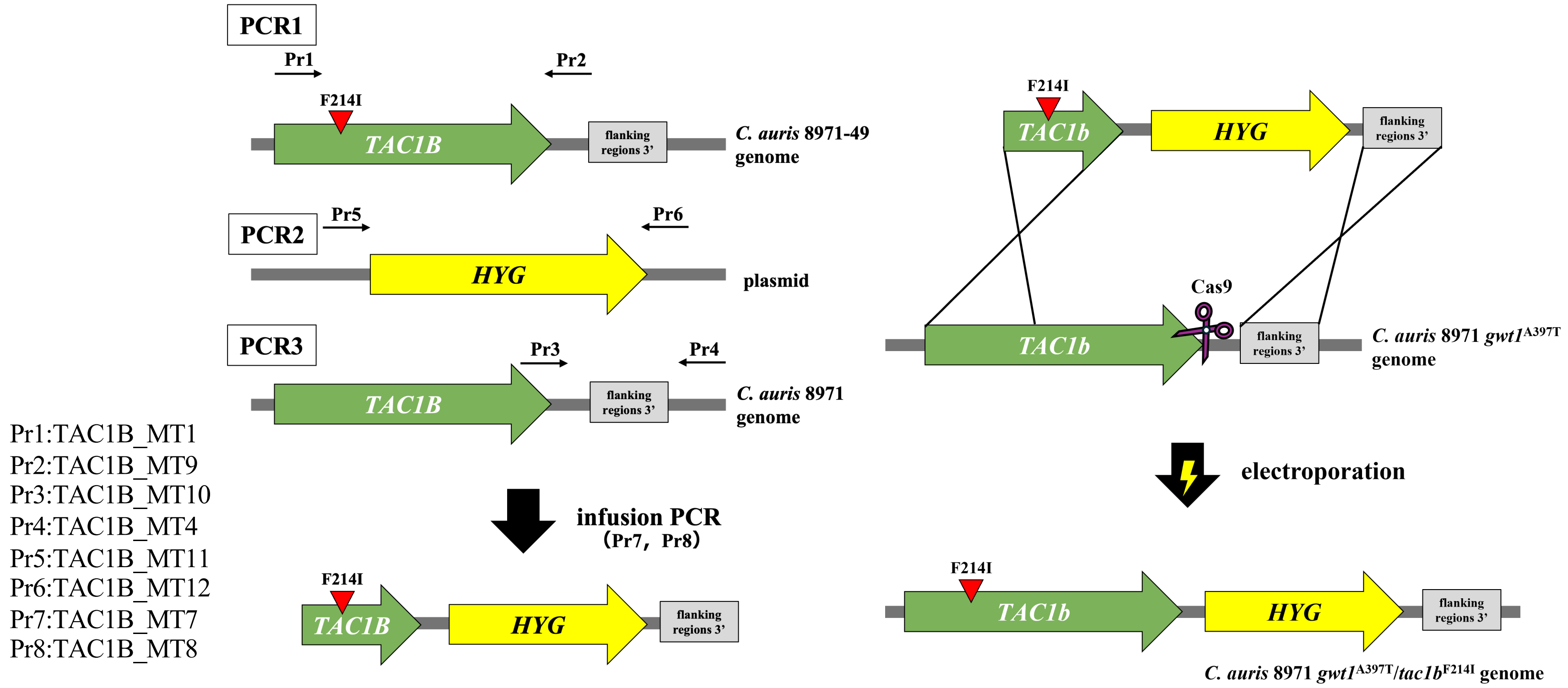

Supplementary Figure S2C. Generation of the *GWT1* A397T/*TAC1B* F214I double mutant by CRISPR-Cas9.

This figure is a schematic diagram illustrating the procedure for generating *GWT1*/*TAC1B* mutant lines. The hygromycin resistance marker (*HYG*) was used.
