# Supplementary material for: A novel GWT1 A397T substitution combined with TAC1B F214I reduces manogepix susceptibility and fosmanogepix efficacy in Candida auris
Source: Microbiol Spectr. 2026 Sep 1;14(10):e02078-26. doi: 10.1128/spectrum.02078-26 (PMC13688795; doi:10.1128/spectrum.02078-26)
Supplement: Supplementary file 2 [file spectrum.02078-26-s0001.docx]

**Supplementary Table S1. The MICs of 94 isolates from YPD agar containing manogepix**

| **Manogepix MIC (μg/ml)** | **0.03** | **0.06** | **0.125** | **0.25** | **0.5** |
| --- | --- | --- | --- | --- | --- |
| **Number of isolates** | **7** | **47** | **18** | **9** | **13** |

**Supplementary Table S2. The primers used in this study**

| For gene sequencing | | |
| --- | --- | --- |
| Aim | Primer | Sequence (5’-3’) |
| *GWT1* sequencing | GWT1-F | TCGTAGTAACCTTCGGACC |
|  | GWT1-R | GCACTCGTTTTCTGATGTC |
|  | GWT1-F-74 | CGTAACCCTCTTTTGCTATGG |
|  | GWT1-F600 | GCTTCTACACAACACCCTG |
| *TAC1B* sequencing | TAC1B-F | AACCCGCTTACTCATCATC |
|  | TAC1B-R | GGAGAGAGTGTCTAAGGACC |
|  | TAC1B-F-356 | ATTACGATGATGCGTTCCC |
|  | TAC1B-F379 | AGCAACAACAACGCTTCC |
|  | TAC1bBF1216 | GAACTTGGTCTTCACAGATTC |
|  | TAC1B-F1952 | CCATGATGAACTTGATTGC |
| For introduction of *GWT1*^A397T^ mutation | | |
| *GWT1* mutation introduction | GWT1_MT1 | AGACTGAACCTCCCAAAGAG |
|  | GWT1_MT2 | TGACCCGGCGGGGACGAGGCAAGCTTTAGAGTTTGATGTATATCTTGAACTTG |
|  | GWT1_MT3 | GATACTAACGCCGCCATCCAGTGTCATGGTTGCAAAATGCGACA |
|  | GWT1_MT4 | CTCTGGATGTATGAGGTGCC |
|  | GWT1_MT5 | GTTCAAGATATACATCAAACTCTAAAGCTTGCCTCGTCCCCGCCG |
|  | GWT1_MT6 | TTCTGATGTCGCATTTTGCAACCATGACACTGGATGGCGGCGTTAGTATC |
|  | GWT1_MT7 | CAAGTCCTTCATCACAGCG |
|  | GWT1_MT8 | CAGTGATAAGCCCCTCAATG |
|  | GWT1_MTgRNA1 | CATTGGACCTCTTAAGCTGA |
|  | GWT1_MTgRNA2 | GCTGACCACCCAGAGTACAT |
| For introduction of *TAC1B*^F214I^ mutation | | |
| *TAC1B* mutation introduction | TAC1B_MT1 | GAAGGGCAAAAGAGACAGG |
|  | TAC1B_MT2 | TGACCCGGCGGGGACGAGGCAAGCTCATACTATTTACAAAGTTAAAGCCC |
|  | TAC1B_MT3 | GATACTAACGCCGCCATCCAGTGTCCTTACCACGATTTTAACGATGA |
|  | TAC1B_MT4 | AGGCTATTGCAAAGTACGACC |
|  | TAC1B_MT5 | GGGCTTTAACTTTGTAAATAGTATGAGCTTGCCTCGTCCCCGCCG |
|  | TAC1B_MT6 | TCATCATCGTTAAAATCGTGGTAAGGACACTGGATGGCGGCGTTAGTATC |
|  | TAC1B_MT7 | CGCTTTTACTTCGAGCCTG |
|  | TAC1B_MT8 | ATGGGAGCTTTACCAGGATATC |
|  | TAC1B_MT9 | CGGGGAAATGTGCGCGGAACCCCTACATACTATTTACAAAGTTAAAGCCC |
|  | TAC1B_MT10 | TATTTTCGTCACGGGTATTTTCTCTCTTACCACGATTTTAACGATGA |
|  | TAC1B_MT11 | GGGCTTTAACTTTGTAAATAGTATGTAGGGGTTCCGCGCACATTTC |
|  | TAC1B_MT12 | TCATCATCGTTAAAATCGTGGTAAGAGAGAAAATACCCGTGACGAAAATAATAAAATAAG |
|  | TAC1B_MTgRNA1 | TGAATCCAGGCTCAAGCCAG |
|  | TAC1B_MTgRNA2 | CATTACAAGGGATTGCATCT |
